# Effects of Baicalein Pretreatment on the NLRP3/GSDMD Pyroptosis Pathway and Neuronal Injury in Pilocarpine-Induced Status Epilepticus in the Mice

**DOI:** 10.1523/ENEURO.0319-24.2024

**Published:** 2025-01-03

**Authors:** Junling Kang, Shenshen Mo, Xiuqiong Shu, Shuang Cheng

**Affiliations:** Department of Neurology, The Third Affiliated Hospital of Zhejiang University of Chinese Medicine, Hangzhou 310005, China

**Keywords:** baicalein, neuroinflammation, neuronal injury, NOD-, LRR-, and pyrin domain-containing 3/gasdermin-D pathway, pyroptosis, status epilepticus

## Abstract

Status epilepticus (SE) links to high mortality and morbidity. Considering the neuroprotective property of baicalein (BA), we investigated its effects on post-SE neuronal injury via the NLRP3/GSDMD pathway. Mice were subjected to SE modeling and BA interference, with seizure severity and learning and memory abilities evaluated. The histological changes, neurological injury and neuron-specific enolase (NSE)-positive cell number in hippocampal CA1 region, and cell death were assessed. Levels of the NOD-, LRR-, and pyrin domain-containing 3 (NLRP3)/gasdermin-D (GSDMD) pathway-related proteins, inflammatory factors, and Iba-1 + NLRP3+ and Iba-1 + GSDMD-N+ cells were determined. BA ameliorated post-SE cognitive dysfunction and neuronal injury in mice, as evidenced by shortened escape latency, increased number of crossing the target quadrant within 60 s and the time staying in the target quadrant, alleviated hippocampal damage, increased viable cell number, decreased neuronal injury, and increased NSE-positive cells. Mechanistically, BA repressed microglial pyroptosis, reduced inflammatory factor release, and attenuated neuronal injury by inhibiting the NLRP3/GSDMD pathway. The NLRP3 inhibitor exerted similar effects as BA on SE mice, while the NLRP3 activator partially reversed BA-improved post-SE neuronal injury in mice. Conjointly, BA reduced microglial pyroptosis in hippocampal CA1 area by inhibiting the NLRP3/GSDMD pyroptosis pathway, thereby ameliorating post-SE neuronal injury in mice.

## Significance Statement

This study highlights the ameliorative role of BA in neuronal injury via repression of the NLRP3/GSDMD pyroptosis pathway in SE mice, offering theoretical support for the treatment of SE using BA.

## Introduction

Seizures represent a chronic condition that is manifested as transient brain dysfunction triggered by sudden abnormal discharges of brain neurons (Thijs et al., 2019). Status epilepticus (SE) is a persistent and fixed state featured by frequent or sustained seizure (Meng et al., 2014). Notably, if not treated in time, SE can cause irreversible brain damage, including neuronal death, neuronal injury, and changes in neuronal networks (Trinka et al., 2015). Currently, pharmacological treatment is recognized as the main and the most effective means of antiepileptic treatment, and traditional low-cost antiepileptic medications include benzodiazepines, carbamazepine, and valproic acid, whereas the majority of antiepileptic drugs have been proved to generate apparent toxic side effects (Abou-Khalil, 2016). Moreover, nonpharmacological treatment for seizures, such as surgery and dietary therapy, has been developed greatly in developed countries (Evans et al., 2012; Cervenka et al., 2016), but due to the low-income situation of the main epileptic population, most epileptic patients still choose pharmacological treatment.

Baicalein (BA), extracted from *Scutellaria* root, stands out as one of the main active components for *Scutellaria baicalensis* to exert its efficacy, which has the properties of anti-inflammation, antioxidation, and neuroprotection (Sowndhararajan et al., 2017; B. Li et al., 2021; Song et al., 2021). BA has been reported to have positive effects on neurological disorders in various studies. For instance, BA contributes to the alleviation of ischemic brain injury by repressing microglia polarization and ferroptosis (Ran et al., 2021; Li et al., 2022). Also, BA is able to assuage hippocampal damage and cognitive impairment in rats with temporal lobe seizures (Qian et al., 2019). It is already known that BA has antiseizure activity, but its specific mechanism of antiseizure effect remains unclear.

Various clinical and basic research data have implied that neuroinflammation plays an essential role in SE (Tan et al., 2015; Mohseni-Moghaddam et al., 2021; Xia et al., 2021a). On the one hand, inflammation stimulates the release of proinflammatory cytokines, which in turn raises the incidence of SE, and on the other hand, SE episodes induce an influx of inflammatory molecules and cause neuroinflammation (Vezzani et al., 2011). Furthermore, once SE develops, it enables the persistent existence of inflammation in the brain, thereby activating a vicious cycle, which in turn promotes abnormal neuronal hyperexcitability and leads to a long-term condition of persistent seizures (Meng et al., 2014).

Pyroptosis represents a programmed cell death resulting from the inflammasomes (Kovacs and Miao, 2017). NOD-, LRR-, and pyrin domain-containing 3 (NLRP3) stands out as the most typical inflammasomes that are composed of caspase-1 and apoptosis-associated speck-like protein containing CARD (ASC; Kovacs and Miao, 2017; Fu and Wu, 2023). Gasdermin (GSDM) protein is a key effector molecule in cell pyroptosis, with the mechanism by which GSDMD induces cell pyroptosis relatively clear (Liu et al., 2021). When stimulated, NLRP3 leads to caspase-1 activation, which, on the one hand, cleaves GSDMD proteins to form peptides comprising GSDMN active domains and induces perforation of cell membranes, cell disruption, and release of contents, ultimately resulting in cell death (Guo et al., 2015; Fang et al., 2020; Akbal et al., 2022). On the other hand, the activated caspase-1 cleaves interleukin (IL)-18 and IL-1β precursors to form and release active IL-18 and IL-1β extracellularly, thereby recruiting inflammatory cells and aggravating the inflammatory response (Schroder and Tschopp, 2010; Guey et al., 2014). Increased concentration of intracellular Ca^+^, production of reactive oxygen species (ROS), and concentrations of extracellular ATP and K^+^ are all implicated in activating the NLRP3 inflammasomes (Wang et al., 2019). Notably, activated NLRP3 advances caspase-1 activation, pyroptosis, and inflammation, thereby contributing to the occurrence and development of seizures (Wu et al., 2019). Besides, Rui et al. (2020) have identified that BA plays a mitigative role in neuroinflammation in mice with Parkinson's disease through dampening the NLRP3/GSDMD pathway. However, it remains unclear whether BA has the inhibitory effect on the NLRP3/GSDMD pyroptosis pathway in mice after SE.

Microglia are the inherent immune effector cells in the central nervous system, which is also known as the crucial effector cells for the nervous system primary disease or infection-triggered inflammation (Nayak et al., 2014; Xu et al., 2016). When neurological disorders (such as trauma, infection, inflammation) happen in the brain, microglia are rapidly activated and obtain phagocytic function (Kwon and Koh, 2020). Reportedly, agmatine has been proved to curb the activation of the NLRP3/GSDMD pathway in microglia and lighten epileptic seizures in vivo (X. Li et al., 2021), suggesting that the improvement of epileptic condition may be achieved by the inactivation of the microglial NLRP3/GSDMD pathway. Nevertheless, whether BA can suppress the microglial NLRP3/GSDMD pyroptosis pathway and relieve post-SE neuronal injury in mice remains largely unknown. As a consequence, this study aimed to explore the mechanism, with the aim of offering theoretical support for the treatment of SE using BA.

## Material and Methods

### Ethics statement

Animal experiments were reviewed and authorized by the Animal Ethics Committee of The Third Affiliated Hospital of Zhejiang University of Chinese Medicine. All procedures were carried out in strict accordance with the code of ethics. We had done our utmost to minimize the animal amount and utilized all laboratory procedures to minimize the pain of the mice, including heating pads, sterilization, and fluid supplementation with saline.

### Establishment of SE mouse model

A total of 84 male C57BL/6J mice (8 weeks old) were procured from Zhejiang Research Institute of Traditional Chinese Medicine [SYXK (Zhejiang) 2023-0025]. The mice were fed in separate cages at 20–26°C, with *ad libitum* access to food and water, and in a 12 h light/dark cycle.

Following a week of adaptive feeding, an SE mouse model was established by intraperitoneal injection with pilocarpine. Briefly, mice were injected intraperitoneally with 1 mg/kg atropine (Sigma-Aldrich) 30 min before administration to eliminate the peripheral response brought about by pilocarpine, followed by intraperitoneal injection of 300 mg/kg pilocarpine (Sigma-Aldrich). Subsequently, a researcher who was unaware of the experimental conditions recorded the behavioral status of the mice every 10 min after the onset of the seizure response utilizing a modified Racine scale (stage 0, no seizure activity/normal behavior; stage 1, freezing; stage 2, single twitches; stage 3, orofacial seizures; stage 4, clonic seizures; stage 5, tonic seizures; stage 6, death; Lenz et al., 2019). The establishment of SE mouse modeling was considered successful when seizures reached stages 4–5 and lasted up to 60 min. Behavioral assessment was terminated immediately after 60 min SE, and then the mice underwent intraperitoneal injection with 5 mg/kg diazepam (Sigma-Aldrich) to terminate SE progression.

### Experimental grouping and drug treatment

The 84 mice were randomly allocated into the following seven groups (*n* = 12): the CON group (normal mice); the CON + BA group (injected only with 40 mg/kg BA); the SE group (model mice); the SE + BA group (intraperitoneally injected with 40 mg/kg BA once a day for 2 weeks prior to SE induction; Mao et al., 2014); the SE + MCC950 group (intraperitoneally injected with 50 mg/kg MCC950 30 min before the injection of pilocarpine and administered the same dose of MCC950 again 6 h after the induction of SE; Yue et al., 2020); the SE + DMSO group [treated with dimethyl sulfoxide (DMSO) at the same dose as MCC950 and subjected to other treatments same as the SE + MCC950 group]; the SE + BA + NS group [injected with BA and intraperitoneally injected three times with 4 mg/kg nigericin sodium (NS) every other day before SE induction; Deng et al., 2013]; and SE + BA + DMSO group (treated with DMSO at the same dose as NS and subjected to other treatments consistent with the SE + BA + NS group). BA (95%; Sigma-Aldrich) was dissolved in saline, and MCC950 (97%; Sigma-Aldrich) and NS (98%; Solarbio) were dissolved in DMSO (Sigma-Aldrich). All mice were subjected to a 7 d behavioral assay 12 h following the induction of SE. After the Morris water maze (MWM) test, the mice were anesthetized by intraperitoneally injecting with 50 mg/kg of 1% sodium pentobarbital, transcardially perfused with 4% paraformaldehyde and saline, and then killed. Subsequently, mice were collected for the hippocampal CA1 region, with that from six mice applied for histological assay and that from the other six mice employed for Western blot, lactate dehydrogenase (LDH) assay, and enzyme-linked immunosorbent assay (ELISA).

### MWM test

The MWM test, consisting of spatial probe test and place navigation test, reflects memory and learning abilities of mice. It required a pool (120 cm in diameter, 50 cm in depth) filled with water at 21°C and a platform (10 cm in diameter, 20–35 cm in height), with the water surface 1 cm above the platform. The pool was equally separated into four quadrants as per the north, south, east, and west directions (NE, SE, SW, and NW), with the platform placed in the center of any quadrant. The place navigation test lasted for 5 d. Prior to the test, the mice were placed in the pool and allowed to swim freely for 2 min to familiarize themselves with the maze environment, and the test was conducted four times at the same time period every day. The mice were released into the pool facing the wall from any point of the four quadrants, with the time they required to find the platform within 60 s (escape latency) recorded. If the mice failed to find the platform within 60 s, they were guided to the platform and allowed to stay on the platform for 10 s, and the escape latency was recorded as 60 s. The interval between the two training sessions was 15 min, and the mice were wiped dry and placed back to their cages at the end of the experiment. Then, the 60 s spatial probe test began 24 h after the end of the place navigation test. After the platform was removed, the mice were released into the pool from the opposite quadrant of the original platform. Finally, the swimming time of the mice in the quadrant where the original platform was located and the number of times they crossed the area where the original platform was located were recorded with the aid of the SuperMaze software (Xinruan Information Technology).

### Extraction of mouse hippocampal tissues

All mice were killed, and then their head was severed with scissors from the foramen magnum of the occipital bone. The bone at the top of the skull was carefully clipped off using vascular forceps along the foramen magnum of the occipital bone to fully expose the brain. Thereafter, the nerves of the base of the skull were carefully and obtusely peeled off utilizing ophthalmic forceps, and the whole brain tissues were taken out and put into the glass dish lined with tinfoil at 4°C, followed by the subsequent operations conducted on ice. The brain tissues were obtusely detached from the outer cleft utilizing two ophthalmic forceps, and the crescent-shaped hippocampal tissues were visible.

### Hematoxylin and eosin staining

Hippocampal tissues were gathered and subjected to a 24 h fixation with 4% paraformaldehyde (Solarbio), alcohol dehydration, xylene clear, paraffin embedding, sectioning at 5 μm, and dewaxing to water. Then, the samples were stained with the HE staining kit (G1120, Solarbio), dehydrated with gradient alcohol and sealed with neutral gum, followed by observation and photographing under a Nikon Ti optical microscope.

### Nissl staining

After staining with 0.5% cresyl violet (Sigma-Aldrich) for 10 min at room temperature, the paraffin sections underwent treatment with 0.25% glacial acetic acid (Sigma-Aldrich) for a few seconds, followed by dehydration with gradient alcohol and xylene (Sigma-Aldrich) clearing. Ultimately, sections were observed and imaged under a light microscope.

### Immunohistochemistry analysis

Hippocampal tissues were collected and fixed in 4% paraformaldehyde for 24 h. Subsequently, the tissues were transferred sequentially into 10, 20, and 30% sucrose solutions in phosphate-buffered saline (PBS) at 4°C until they settled. Hippocampal tissues were sectioned at 2 μm thickness using a cryosectioner (CM1950, Leica), carefully rinsed with PBS, and incubated with 0.3% H_2_O_2_ solution for 10 min. Thereafter, the sections were blocked with 5% goat serum (Solarbio) for 15 min and incubated at 4°C overnight with primary antibody rabbit antineuron-specific enolase (NSE) antibody (1:100, SAB4500768, Sigma-Aldrich), followed by incubation with goat anti-rabbit immunoglobulin G (IgG) H&L (HRP; 1:1,000, ab6721, Abcam) at room temperature for 2 h. Thereafter, the sections were subjected to color development with 3,3-diaminobenzidine (Solarbio), followed by restaining and sealing. The samples were finally observed and photographed under a light microscope. The ImageJ software (National Institutes of Health) was used for cell counting.

### LDH assay

An LDH activity assay kit (Solarbio, BC0685) was employed for the determination of LDH level in hippocampal tissues to evaluate cell death as per the manufacturer's protocol. All experiments were repeated thrice.

### Western blot

Total protein of hippocampal tissues was extracted with the help of a tissue protein extraction kit (P0033, Beyotime), followed by protein concentration determination utilizing a bicinchoninic acid protein concentration assay kit (P0009, Beyotime). The protein (50 μg) was subjected to 10% sodium dodecyl sulfate-polyacrylamide gel electrophoresis separation and then transferred onto a polyvinylidene fluoride membrane at room temperature. Thereafter, the membrane underwent a 1 h blockade with 5% skim milk and an overnight incubation with primary antibodies rabbit anti-NLRP3 antibody (1:1,000, ab270449, Abcam), rabbit anti-GSDMD-N antibody (1:1,000, DF12275, Affinity Biosciences), rabbit anti-cleaved caspase-1 antibody (1:500, ab32042, Abcam), and rabbit anti-apoptosis-associated speck-like protein (ASC) antibody (1:500, AB3607, Sigma-Aldrich) at 4°C, followed by washes with Tris-buffered saline with Tween 20. The samples were then subjected to incubation with goat anti-rabbit IgG H&L (HRP; 1:3,000, ab6721, Abcam) at room temperature for 1 h and then development utilizing the enhanced chemiluminescence working solution (Solarbio). The gray scale of bands was quantified using Image Pro Plus 6.0 (Media Cybernetics), with rabbit anti-β-actin (1:2,000, ab8227, Abcam) serving as an internal reference. All experiments were repeated three times.

### ELISA

Levels of IL-18 and IL-1β in hippocampal tissues were determined using mouse IL-18 ELISA Kit (Solarbio, SEKM-0019) and mouse IL-1β ELISA Kit (Solarbio, SEKM-0002) based on the manufacturer's instructions. Three repetitions were guaranteed for all experiments.

### Immunofluorescence staining

Frozen tissue sections were prepared and then blocked with immunostaining blocking solution (Beyotime) for 30 min. Rabbit anti-NLRP3 antibody (1:50, ab270449, Abcam) and rabbit anti-GSDMD-N antibody (1:200, DF12275, Affinity Biosciences) were mixed with rabbit anti-Iba-1 antibody (1:100, ab178847, Abcam), respectively, and then incubated with the sections at 4°C overnight. Subsequently, the sections were incubated at room temperature with secondary donkey anti-rabbit IgG H&L (Alexa Fluor 647; 1:200, ab150063, Abcam) for 1 h, followed by nuclei restaining with 4′,6-diamidino-2-phenylindole (Solarbio, C0065) for 10 min. Ultimately, images were captured using a fluorescence microscopy (DMI8, Leica), and cell counting was performed using ImageJ 1.48 software (National Institutes of Health).

### Statistical analysis

All data were statistically analyzed and graphed using GraphPad Prism 8.01 software (GraphPad Software). The outcomes were represented as mean ± standard deviation and tested for normality and homogeneity of variance. Data consistent with normal distribution and homogeneity of variance were compared between groups utilizing one-way analysis of variance (ANOVA), with Tukey's multiple-comparisons test used for post hoc tests. The difference was statistically significant at *p* < 0.05.

## Results

### BA relieved post-SE seizure and neuronal injury in mice

**Figure 1.**
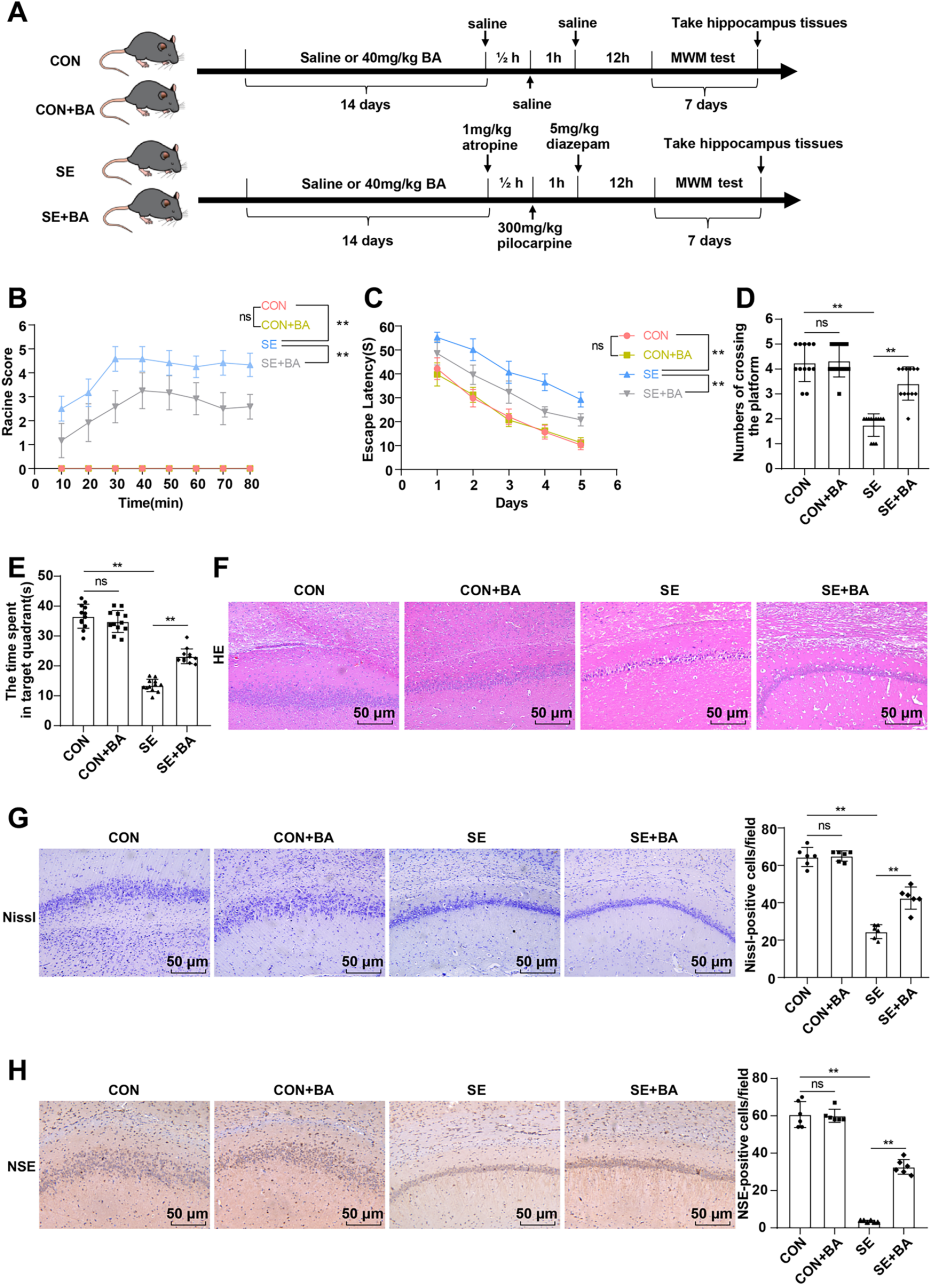
BA-improved post-SE seizure and neuronal injury in mice. Mouse SE model was established by intraperitoneal injection of pilocarpine. ***A***, Animal experiment flow chart; ***B***, Racine scale score, *n* = 12. ***C–E***, MWM test to assess learning and memory abilities of mice, *n* = 12. ***F***, HE staining to observe the histological changes in hippocampal CA1 area. ***G***, The neuronal injury in hippocampal CA1 area was observed by Nissl staining, *n* = 6. ***H***, The number of NSE-positive cells in the hippocampal CA1 area was determined by immunohistochemistry, *n* = 6. The data were denoted as the mean ± standard deviation. One-way ANOVA was used for intergroup comparisons, with Tukey's multiple-comparisons test applied for post hoc tests. ^ns^*p* > 0.05, **p < 0.01.

BA possesses the capacity to attenuate hippocampal tissue damage and seizure in mice (Qian et al., 2019; Fu et al., 2020), but it has not been clarified with regard to the mechanism of BA on post-SE neuronal injury in mice. We established an SE mouse model and used BA to intervene with it (Fig. 1*A*). The Racine scale (Fig. 1*B*) evaluating the severity of seizures in mice unveiled a notable elevation in the Racine score of SE mice compared with that of mice in the CON group, whereas the intervention of BA apparently alleviated the post-SE seizure of mice (all *p* < 0.01). The MWM evaluating learning and memory abilities of mice disclosed a remarkable extension in the escape latency of SE mice relative to that of mice in the CON group (Fig. 1*C*), an obvious reduction in the number of times they crossed the target quadrant within 60 s (Fig. 1*D*), and a noticeable decrease in the time they spent in the target quadrant (all *p* < 0.01; Fig. 1*E*), whereas BA intervention ameliorated the behavioral abnormalities of SE mice (all *p* < 0.01; Fig. 1*C–E*). After completion of the MWM test, the hippocampal CA1 region was extracted from the mice for histological assay. Next, HE staining indicated densely arranged cells and intact cell morphology in the hippocampal region of mice in the CON group and severely damaged hippocampus of the SE mice, with cavities left behind by the loss of cells visible, while BA intervention attenuated the hippocampal tissue damage brought about by SE (Fig. 1*F*). Nissl staining results elicited neuronal atrophy, nuclear atrophy, and nissl body loss in SE mice, along with a markedly reduced number of neurons in SE mice versus mice in the CON group, whereas BA intervention alleviated the neuronal damage of SE mice and increased the number of neurons (*p* < 0.01; Fig. 1*G*). As reflected by immunohistochemistry results, a marked abatement in the level of NSE-positive cells in the hippocampus of SE mice was noted in contrast to that of mice in the CON group, but a significant elevation in the level of NSE-positive cells in the hippocampus of BA-intervened SE mice was then observed (all *p* < 0.01; Fig. 1*H*). Additionally, treatment of the same dose of BA alone brought about no significant adverse effects on mice in the CON group (all *p* > 0.05; Fig. 1*B–H*). The findings suggest that BA can improve neuronal damage following SE in mice.

### BA repressed the activation of the NLRP3/GSDMD pyroptosis pathway and attenuated microglia pyroptosis after SE in mice

**Figure 2.**
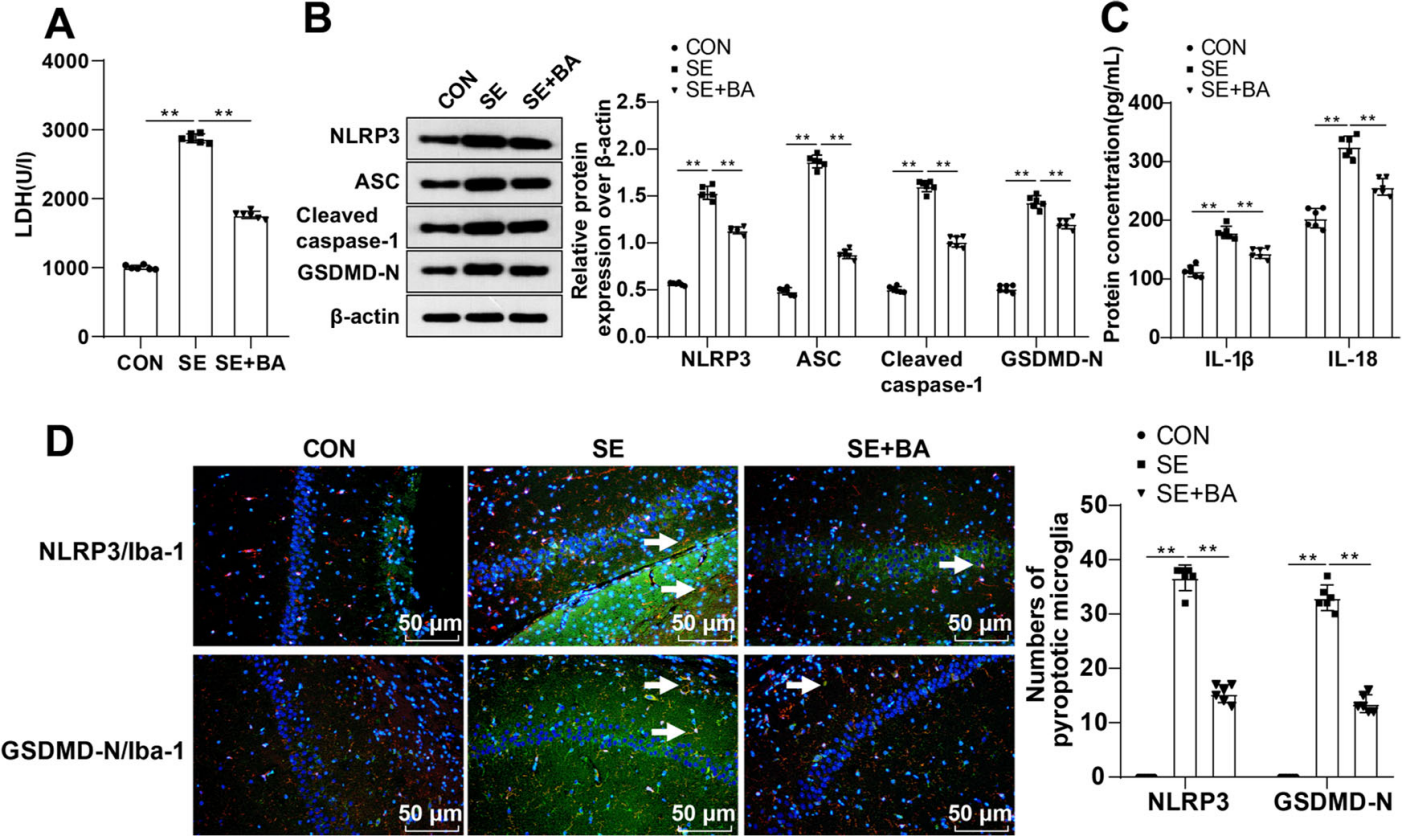
BA limited NLRP3/GSDMD pyroptosis and alleviated microglial pyroptosis following SE in mice. ***A***, LDH level in the hippocampal CA1 area of mice was determined by LDH assay to assess cell death. ***B***, Protein levels of NLRP3, cleaved caspase-1, ASC, and GSDMD-N were assessed by Western blot. ***C***, ELISA to determine levels of IL-18 and IL-1β. ***D***, Levels of Iba-1 + NLRP3+ and Iba-1 + GSDMD-N+ cells were measured by immunofluorescence, and white arrows indicated Iba-1 + NLRP3+ cells or Iba-1 + GSDMD-N+ cells, *n* = 6. Data were presented as mean ± standard deviation. Intergroup comparisons were conducted using one-way ANOVA, followed by Tukey's multiple-comparisons test. **p < 0.01.

It has been evidenced that there is a correlation between the NLRP3 inflammasomes and seizures, and microglial pyroptosis plays a critical role in seizures (Xia et al., 2021b). We further assessed the pyroptosis of microglia in the hippocampal CA1 region of mice after completion of the MWM test. LDH assay evaluating cell death in the hippocampal CA1 region manifested notably higher LDH release in SE mice than mice in the CON group but significantly lowered LDH release in SE mice after BA intervention (all *p* < 0.01; Fig. 2*A*). Western blot assay was conducted to measure levels of the hippocampal NLRP3/GSDMD pathway-associated proteins, which manifested noticeably raised levels of NLRP3, cleaved caspase-1, ASC, and GSDMD-N proteins in SE mice relative to mice in the CON group, while significantly diminished levels were identified in BA-intervened SE mice (all *p* < 0.01; Fig. 2*B*). Moreover, ELISA results indicated obviously increased levels of IL-18 and IL-1β in SE mice versus mice in the CON group, while prominently abated levels were found in BA-intervened SE mice (all *p* < 0.01; Fig. 2*C*). As shown by immunofluorescence detection, Iba-1 + NLRP3+ and Iba-1 + GSDMD+ cells were increased dramatically in SE mice relative to mice in the CON group, while the numbers of Iba-1 + NLRP3+ and Iba-1 + GSDMD+ cells declined remarkably in BA-intervened SE mice (all *p* < 0.01; Fig. 2*D*). These results indicate that BA inhibits the NLRP3/GSDMD pyroptosis pathway and mitigates microglial pyroptosis after SE in mice.

### Restraint of the NLRP3/GSDMD pyroptosis pathway improved post-SE neuronal injury in mice

**Figure 3.**
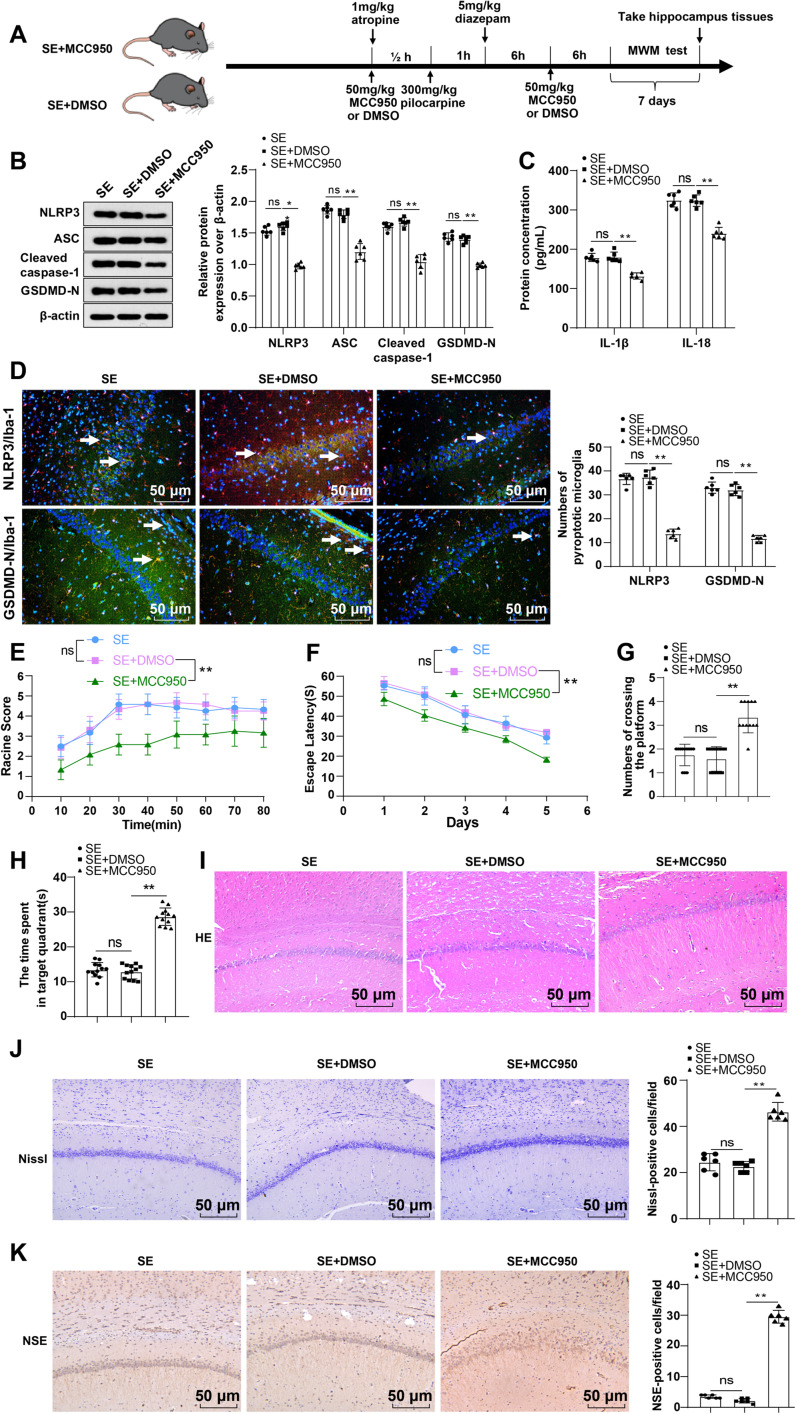
MCC950 dampened neuronal injury in SE mice. ***A***, Animal experiment flow chart. ***B***, Levels of NLRP3, cleaved caspase-1, ASC, and GSDMD-N proteins were measured by Western blot, *n* = 6. ***C***, IL-18 and IL-1β levels were assayed by ELISA, *n* = 6. ***D***, Iba-1 + NLRP3+ and Iba-1 + GSDMD-N+ cell levels were measured by immunofluorescence, and white arrows indicated Iba-1 + NLRP3+ cells or Iba-1 + GSDMD-N+ cells, *n* = 6. ***E***, Racine scale score, *n* = 12. ***F–H***, MWM to assess learning and memory abilities of mice, *n* = 12. ***I***, Observation of the histological changes in hippocampal CA1 area by HE staining. ***J***, Observation of the nerve injury in hippocampal CA1 area by Nissl staining, *n* = 6. ***K***, The number of NSE-positive cells in the hippocampal CA1 area was assayed by immunohistochemistry, *n* = 6. The data were manifested as the mean ± standard deviation. One-way ANOVA was adopted for intergroup comparisons, followed by Tukey's test. ^ns^*p* > 0.05, *p < 0.05, **p < 0.01.

Next, we treated SE mice with a NLRP3 inhibitor MCC950 (Fig. 3*A*). Compared with the SE + DMSO group, mice in the SE + MCC950 group exhibited significantly suppressed levels of NLRP3, ASC, cleaved caspase-1, and GSDMD-N proteins in the hippocampal CA1 area (all *p* < 0.01; Fig. 3*B*), evidently diminished expression levels of IL-18 and IL-1β (all *p* < 0.01; Fig. 3*C*), notably reduced numbers of Iba-1 + NLRP3+ and Iba-1 + GSDMD-N+ cells (all *p* < 0.01; Fig. 3*D*), an obviously decreased Racine score (*p* < 0.01; Fig. 3*E*), a saliently shortened escape latency, a remarkably increased number of times traversing the target quadrant within 60 s and a significant prolonged duration of staying in the target quadrant (all *p* < 0.01; Fig. 3*F–H*), obviously abated hippocampal CA1 area tissue damage and neuronal injury (*p* < 0.01; Fig. 3*I,J*), and an augmented number of NSE-positive cells (*p* < 0.01; Fig. 3*K*). These results suggest that inhibiting the NLRP3/GSDMD pathway can effectively alleviate neuronal injury after SE in mice.

### Activation of the NLRP3/GSDMD pathway partially counteracted the ameliorative role of BA in post-SE microglial pyroptosis in mice

**Figure 4.**
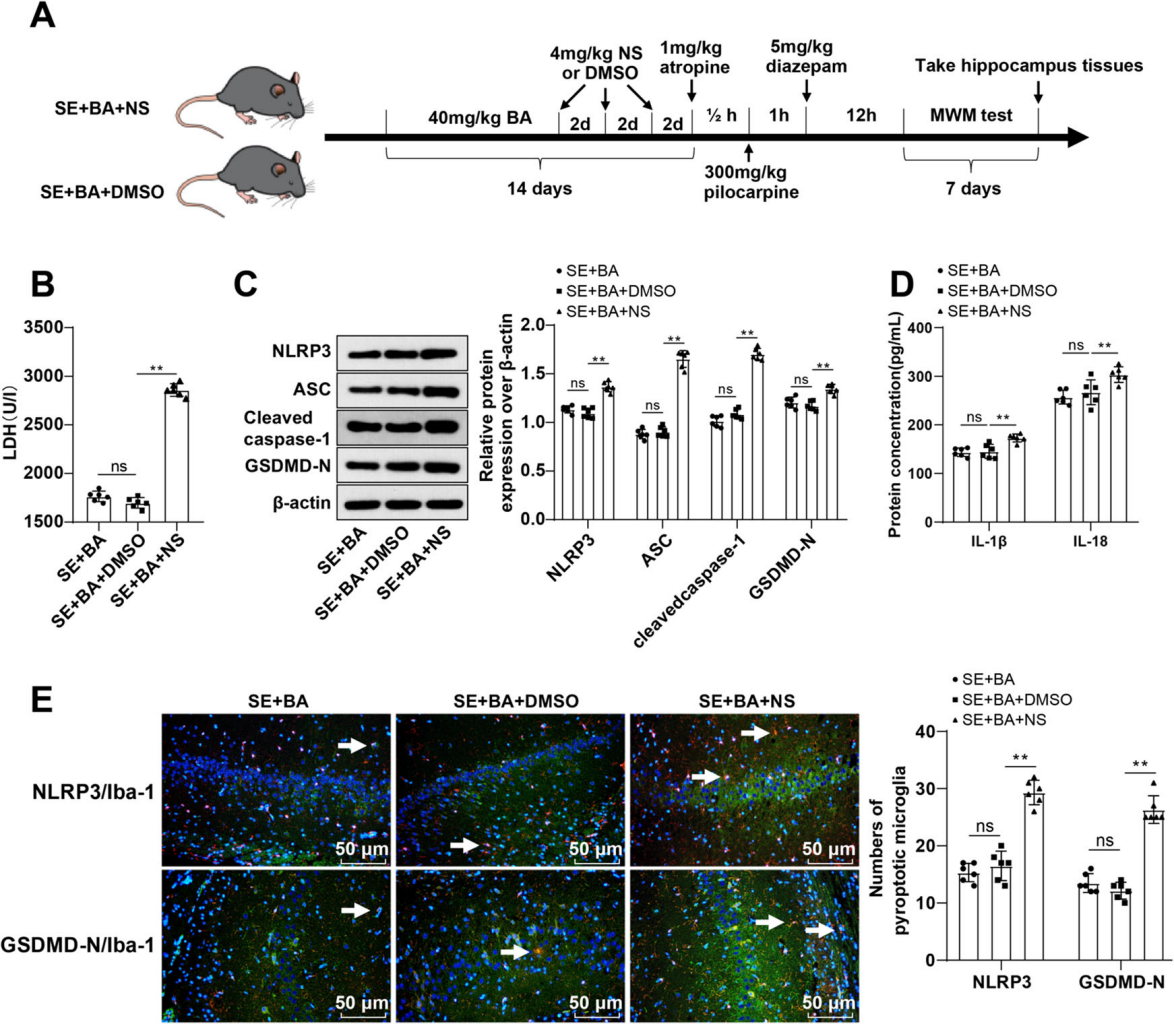
Activation of the NLRP3/GSDMD pyroptosis pathway partially abrogated the ameliorative effect of BA on post-SE microglial pyroptosis in mice. ***A***, Animal experiment flow chart. ***B***, LDH level in the hippocampal CA1 area was determined for cell death assessment. ***C***, Western blot to measure the protein levels of NLRP3, ASC, cleaved caspase-1, and GSDMD-N. ***D***, IL-18 and IL-1β levels were measured by ELISA. ***E***, Iba-1 + NLRP3+ and Iba-1 + GSDMD-N+ cell levels were determined by immunofluorescence, and white arrows indicated Iba-1 + NLRP3+ cells or Iba-1 + GSDMD-N+ cells, *n* = 6. Data were presented as mean ± standard deviation. Intergroup comparisons were accomplished by one-way ANOVA, with Tukey's test performed for post hoc tests. ^ns^*p* > 0.05, **p < 0.01.

Subsequently, we used a NLRP3 activator NS to treat SE mice upon BA treatment (Fig. 4*A*). Compared with those in the SE + BA + DMSO group, mice in the SE + BA + NS group displayed dramatically augmented LDH release in the hippocampal tissues (*p* < 0.01; Fig. 4*B*), significantly elevated levels of NLRP3, ASC, cleaved caspase-1 and GSDMD-N proteins, and the inflammatory factors IL-18 and IL-1β (all *p* < 0.01; Fig. 4*C*,*D*); and significantly heightened levels of Iba-1 + NLRP3+ and Iba-1 + GSDMD-N+ cells (all *p* < 0.01; Fig. 4*E*). Taken together, activation of the NLRP3/GSDMD pathway partially annuls the mitigatory effect of BA on microglial pyroptosis in mice after SE.

### BA suppressed post-SE neuronal injury by inhibiting the NLRP3/GSDMD pyroptosis pathway in mice

**Figure 5.**
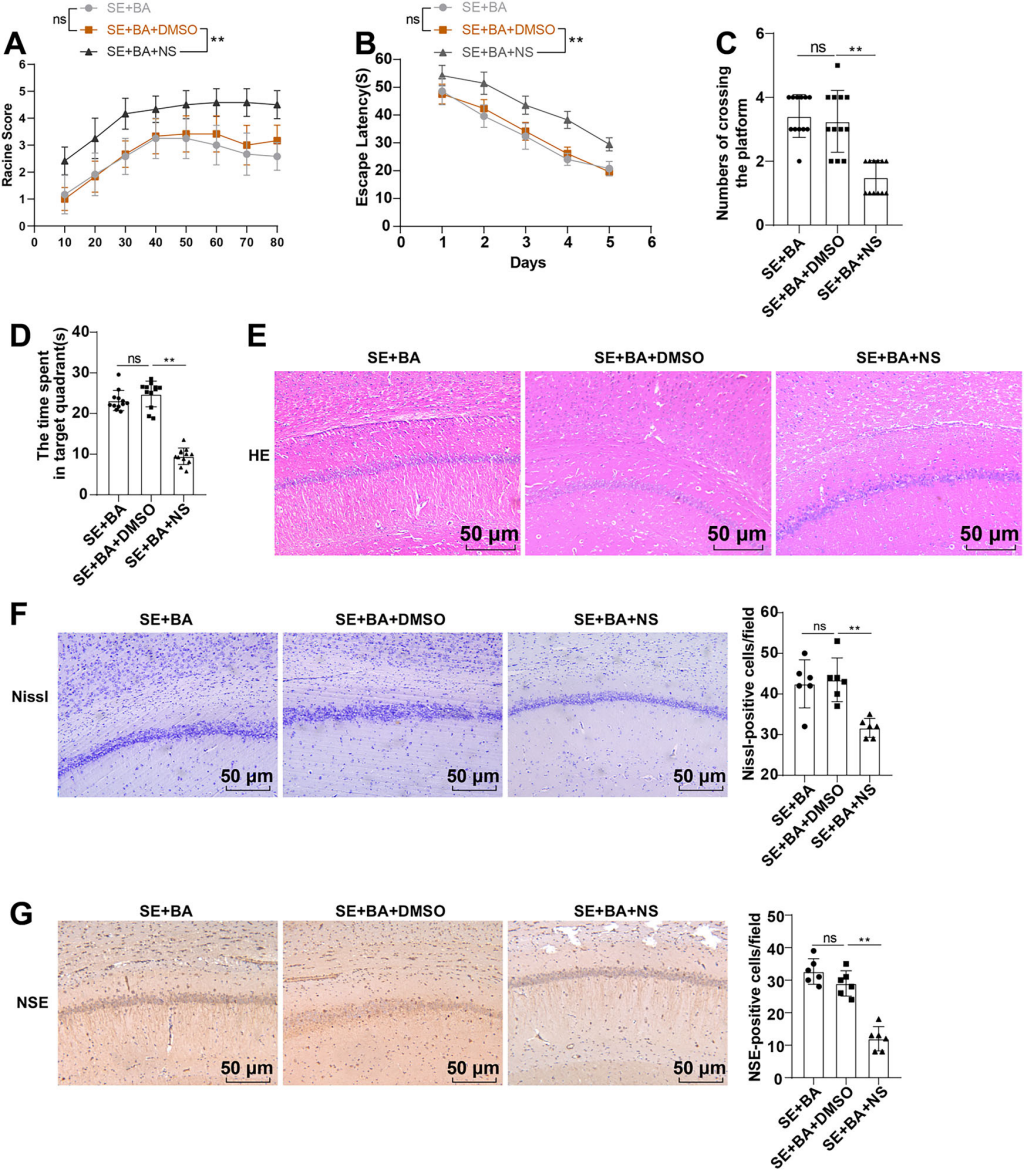
NS partly averted BA-caused amelioration on neuronal injury in SE mice. ***A***, Racine scale score, *n* = 12. ***B–D***, Learning and memory abilities of mice were assessed by MWM test, *n* = 12. ***E***, HE staining to observe the histological changes in hippocampal CA1 area. ***F***, Nissl staining to observe the neuronal injury in hippocampal CA1 area, *n* = 6. ***G***, Immunohistochemistry to determine the number of NSE-positive cells in the hippocampal CA1 area, *n* = 6. The data were expressed as the mean ± standard deviation, which were compared between groups using one-way ANOVA, followed by Tukey's test. ^ns^*p* > 0.05, **p < 0.01.

Finally, we further examined the neuronal injury in mice of the SE + BA, SE + BA + DMSO, and SE + BA + NS groups. The Racine score was prominently higher (Fig. 5*A*), escape latency was apparently longer, the number of times crossing the target quadrant within 60 s was obviously fewer and time spent in the target quadrant was significantly shorter (all *p* < 0.01; Fig. 5*B–D*), hippocampal tissue and neuronal injuries were more severe (all *p* < 0.01; Fig. 5*E,F*), and the number of NSE-positive cells was evidently less (*p* < 0.01; Fig. 5*G*) in mice of the SE + BA + NS group than the SE + BA + DMSO group. These results indicate that BA improves post-SE neuronal injury by repressing the NLRP3/GSDMD pathway in mice.

## Discussion

SE is considered as a condition evoked by the failure of the mechanisms in charge of terminating seizure or by the setup of mechanisms causing anomalous prolongation of seizures (Trinka et al., 2015). Evidence has revealed that BA exhibits a variety of pharmacological features such as neuroprotective, anticancer, antioxidant, anti-inflammatory, cardioprotective, eye protective, as well as hepatoprotective properties (Sowndhararajan et al., 2017). Thus, our findings highlighted that BA mediated an alleviation on neuronal injury via the repression of the NLRP3/GSDMD pyroptosis pathway activation.

SE is a disease that can lead to long-term consequences such as irreversible brain damage, including neuronal injury and neuronal death (Trinka et al., 2015). However, the role of BA in protecting against SE is not fully understood. Thus, our findings indicated worsened seizure symptoms, impaired learning and memory abilities, severely damaged hippocampus, injured neurons, and significantly reduced number of neurons in SE mice relative to mice in the CON group, whereas BA interference brought out the opposite outcomes in SE mice. Importantly, the reductions in inflammatory stimuli and oxidative stress caused by BA in the brain protect against neuronal damage, which can be a mechanism of the hippocampus protection mediated by BA in temporal lobe seizures rats (Qian et al., 2019). Furthermore, Fu et al. (2020) have demonstrated that BA alleviates seizure symptoms in a rat model initiated by pilocarpine through modulating IGF1R. These findings indicate that BA ameliorates post-SE neuronal injury in mice.

There is a substantial body of evidence indicating a strong relation between pyroptosis and SE (Ye et al., 2024). Pyroptosis has been identified as an inflammatory and regulatory cell death, which can be allocated into two pathways (He et al., 2015). In nonclassical pathways, activated caspase-4/5/11 cleave the GSDMD protein, which causes the emergence of pyroptosis, while in classical pathways, the inflammasomes (AIM2, NLRP1, NLRC4, as well as NLRP3) are stimulated by damage-related molecular patterns or pathogen-related molecular patterns, which in turn controls the caspase-1 activation, and encourages the cleavage of GSDMD, with membrane pores formed by the GSDMN domain of GSDMD to propel pyroptosis (McKenzie et al., 2020). Besides, the GSDME-N terminal possesses the activity of forming membrane pores, which is recently perceived as a pyroptosis mediator (Wang et al., 2017). In our study, we observed an increase in LDH release, increments in NLRP3, GSDMD-N, cleaved caspase-1, and ASC protein patterns, augmentations in IL-18 and IL-1β levels, as well as increases in Iba-1 + NLRP3+ and Iba-1 + GSDMD+ cells in SE mice versus mice in the CON group, but these trends were averted after BA treatment. Consistently, a previous study has highlighted the mitigative effect of BA on the brain injury after intracerebral hemorrhage via the suppressions of the NLRP3 inflammasomes and ROS (Chen et al., 2022). Furthermore, Wang et al. (2021) have suggested that BA leads to the amelioration on inflammation and pyroptosis in hyperlipidemic pancreatitis by inhibiting the NLRP3/caspase-1 pathway. BA results in the remission of neuroinflammation through limiting the NLRP3/caspase-1/GSDMD pathway in mice with Parkinson's disease (Rui et al., 2020). Taken together, BA suppresses the NLRP3/GSDMD pyroptosis pathway and alleviates microglia pyroptosis after SE in mice. Further, our findings revealed observably decreased patterns of the NLRP3/GSDMD pathway-related proteins and inflammatory factors, reduced Iba-1 + NLRP3+ and Iba-1 + GSDMD-N+ cells and reduced Racine score, ameliorated learning and memory abilities, alleviated tissue and neuronal injury in the hippocampal CA1 area, as well as increased number of NSE-positive cells in mice following the NLRP3/GSDMD pathway suppression. Not surprisingly, Wang et al. (2021) have found that limiting the NLRP3/caspase-1 pathway brings about pyroptosis and inflammation alleviations in hyperlipidemic pancreatitis via miR-192-5p upregulation and TXNIP inhibition. Also, Huan et al. (2023) have indicated that the Qiji Shujiang granule elicits the mitigation of pyroptosis via repressing the NLRP3/caspase-1 pathway, thereby producing a neuroprotective effect. Altogether, repressing the NLRP3/GSDMD pathway induces attenuation on neuronal injury in SE mice.

To further confirm whether BA ameliorated microglial pyroptosis in SE mice via the NLRP3/GSDMD pathway, we activated the NLRP3/GSDMD pathway on the basis of BA treatment in mice and found increased LDH release, raised level of NLRP3/GSDMD pathway-related proteins and inflammatory factors, and increased Iba-1 + NLRP3+ and Iba-1 + GSDMD-N+ cell levels. Besides, the neuronal injury in SE mice was aggravated. Significantly, the NLRP3-GSDMD signaling activation aggravates pyroptosis led by renal ischemia-reperfusion (Kuang et al., 2024). Agmatine has been revealed to trigger the ameliorations on hippocampal neuronal injury and epileptic seizures via the limitation of GSDMD-shuttled pyroptosis (X. Li et al., 2021). Additionally, apocynin elicits the elevation of neuroblast production and neuronal survival via decrease of hippocampal oxidative injury post seizures (Lee et al., 2018). Therefore, we conclude that activating the NLRP3/GSDMD pathway partially counteracts the ameliorative effect of BA on microglial pyroptosis in SE mice. Furthermore, BA induces neuronal injury amelioration by inhibiting the NLRP3/GSDMD pyroptosis pathway in SE mice.

In conclusion, this study highlights the ameliorative role of BA in neuronal injury via repression of the NLRP3/GSDMD pyroptosis pathway in SE mice. However, this study overlooks the in-depth exploration of the regulatory mechanism of BA on the NLRP3/GSDMD pyroptosis pathway in microglia. As a consequence, subsequent research endeavors should delve into the mechanism of BA regulating the NLRP3/GSDMD pyroptosis pathway in microglia. Moreover, the application of BA was only conducted before SE induction in mice. We will use a design in which BA is treated after pilocarpine-induced SE as our future direction.

## Data Availability

All data generated or analyzed during this study are included in this article. Further enquiries can be directed to the corresponding author.

## Synthesis

Reviewing Editor: Harald Sontheimer, University of Virginia Decisions are customarily a result of the Reviewing Editor and the peer reviewers coming together and discussing their recommendations until a consensus is reached. When revisions are invited, a fact-based synthesis statement explaining their decision and outlining what is needed to prepare a revision will be listed below. The following reviewer(s) agreed to reveal their identity: Ukpong Eyo.

Both reviewers are supportive of this manuscript but both raise some concerns that warrant some revision. Overall, the study was conasidered to be well-conducted and presents significant findings on the neuroprotective effects of BA in SE.

However, significant concerns still remain:

1. Mis-match in the main text and figure labels: This is a huge concern as throughout the manuscript the authors refer to "normal mice" but according to the labels on the. Figures, these are SE treated mice. This makes it very difficult to adequately understand and therefore interpret the results. This seems to be true for virtually all the figures. The authors should thoroughly add carefully appropriately match the descriptions in the results with the labels in the figures.

2. Timepoints for non-seizure scoring studies: It is not clear when the non-seizure assessment studies (e.g. MWM, Western Blot, H&E, Nissl etc) were conducted. This is never stated in the results ad there seems to be a mention of 12h after SE for the MWM in the methods. The method of euthanasia is mentioned but not the timepoint. But for ease of understanding, it would be best to clearly state in the results description when the studies were conducted. This is also true for clear presentation of when the BA treatment occurred. This is mentioned briefly in the Methods but should be presented in the flow of the Results as well (for ease of understanding).

3. Reference to "epilepsy" rather than "seizures": The study several times refers to the paradigm of the results being presented as "epilepsy" or "epilepsy symptoms". But since epilepsy is a condition of spontaneous seizure occurrence and the study does not look at any such condition, its claims could be mis-leading and should be corrected accordingly.

4. Experimental design: This reviewer understands the design of the experiment as described in the Methods to be one in which BA is applied for two weeks before pilocarpine SE treatment. This design make it difficult is more suitable to a prophylactic treatment approach or a therapeutic approach. Moreover, in the current title, this pre-treatment paradigm is not clearly stated. To this reviewer, a design in which BA is treated after pilocarpine SE would be more informative.
